# Case report: Epstein–Barr virus and constrictive pericarditis—An unusual combination

**DOI:** 10.3389/fped.2023.1215928

**Published:** 2023-07-03

**Authors:** Mario Panebianco, Eduard Limonjiani, Roberto Formigari, Veronica Bordonaro, Aurelio Secinaro, Enrico Cetrano, Adriano Carotti, Lorenzo Galletti, Sonia Albanese

**Affiliations:** ^1^ Clinic Area of Fetal Neonatal and Cardiologic Science, Bambino Gesù Children’s Hospital, IRCCS, Rome, Italy; ^2^ Advanced Cardiothoracic Imaging Unit, Bambino Gesù Children’s Hospital, IRCCS, Rome, Italy

**Keywords:** constrictive pericarditis, Epstein–Barr virus, ventricular mechanics, mononucleosis, effusive-constrictive pericarditis

## Abstract

Constrictive pericarditis is a chronic inflammatory process that can lead to heart failure if not diagnosed and treated correctly. Although Epstein–Barr virus (EBV)–related pericarditis is a very rare condition, it should still be considered for a differential diagnosis. We report the case of an 18-year-old male, who was surgically treated for constrictive pericarditis, in which *in situ* hybridization to Epstein–Barr virus-encoded RNA (EBER) probe of the excised pericardium led to the subsequent etiological diagnosis of chronic pericarditis caused by EBV.

## Introduction

Constrictive pericarditis is a chronic inflammatory process characterized by the fusion, thickening, and calcification of two pericardial layers: the fibrous and the serous. In severe cases, it can reach a shell appearance, incarcerating the heart and preventing its normal re-expansion during the diastolic phase, which gradually leads to diastolic heart failure with all the clinical signs and symptoms related to it. The parietal pericardial layer causes the most pronounced constrictive effect on the heart; however, the visceral layer also contributes to this pathologic process. A wide variety of pathogens, chemicals, and physical agents can alter the standard constitution of pericardial layers with different mechanisms, in which they transform pericardial layers into thickened fibrotic layers that lack natural elasticity, which, in turn, lead to deleterious hemodynamic changes. Cardiac involvement, in the form of pericarditis, with Epstein–Barr virus (EBV) infection is quite rare and generally self-limiting, especially in immunocompetent people (1).

## Case presentation

**Figure 1:**
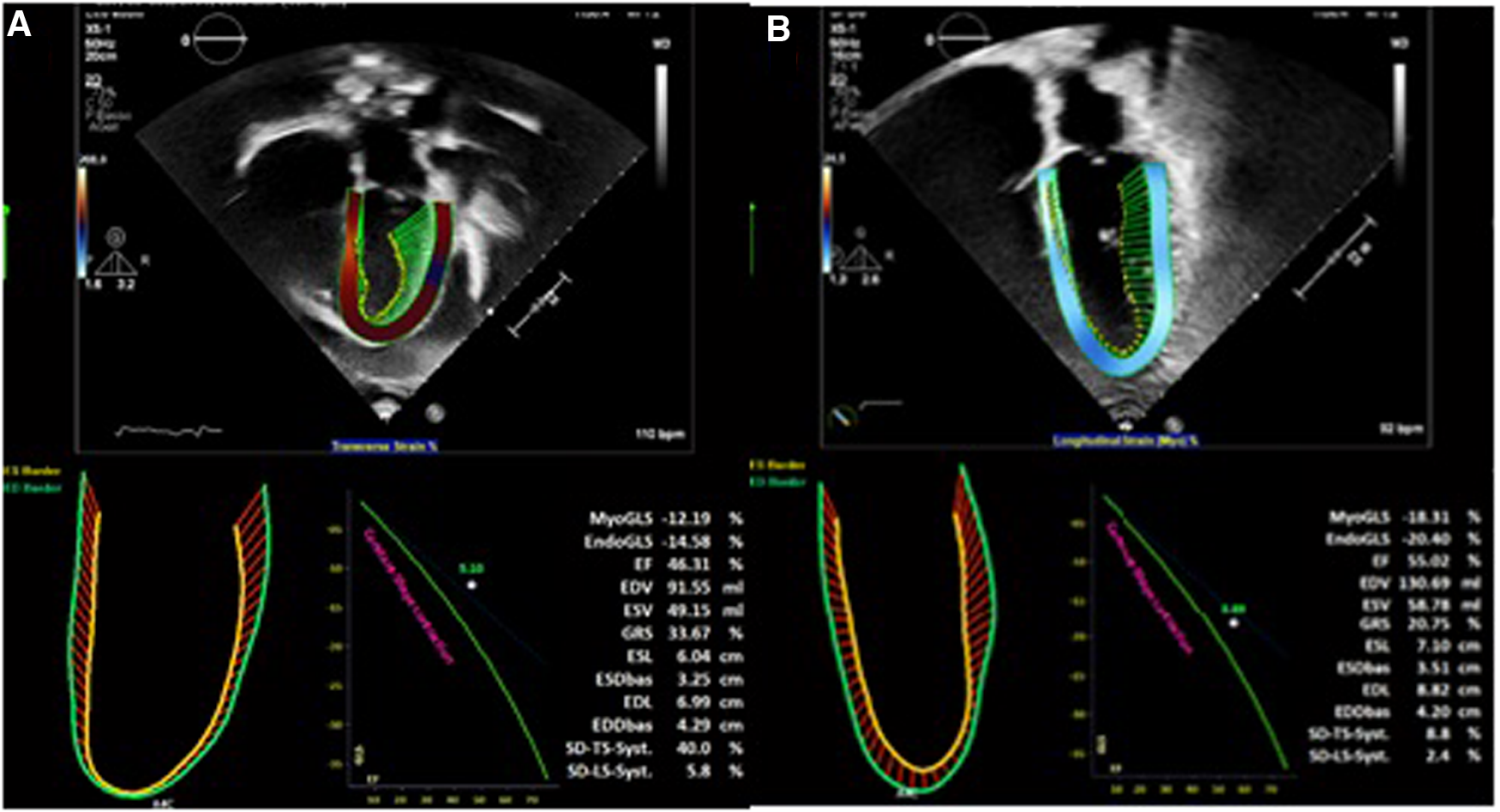
(**A**) Transthoracic echocardiography before cardiac surgery. (**B**) Transthoracic echocardiography after cardiac surgery.

**Figure 2:**
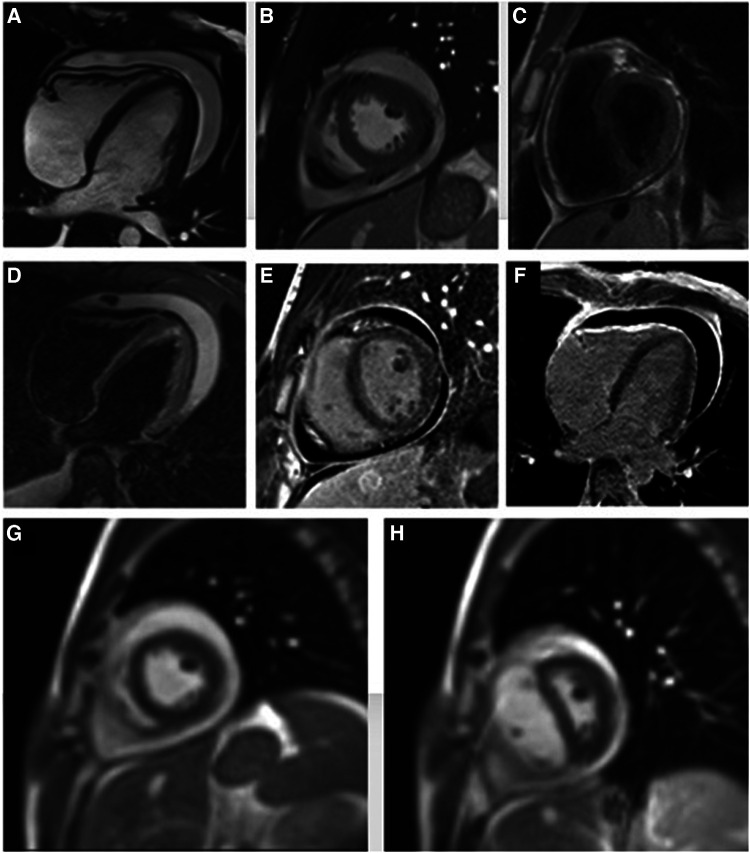
Cine steady-state free precession four- and two-chamber view images show circumferential pericardial effusion (**A,B**), with uniform pericardial thickening at T1-weighted short-axis view image (**C**). There is no significant hyperintense signal on the edema-weighted image (**D**) while a marked late gadolinium enhancement of pericardial layers is evident (**E,F**), suggesting diffuse pericardial fibrosis. Short-axis real-time cine MRI sequence during operator-guided breathing (**G,H**) demonstrates an abnormal displacement of the interventricular septum during the onset of inspiration, due to a fixed pericardial volume with reduced ventricular compliance and consequent increase of the right ventricular filling on inspiration.

**Figure 3:**
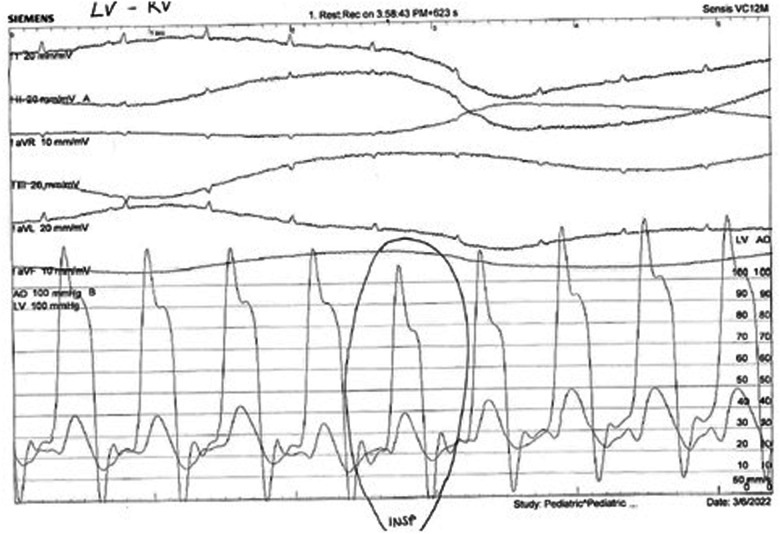
Normally, there is a concordant fall in LV and RV pressures in inspiration. In constriction, there is discordance of LV and RV pressure changes in inspiration: the LV pressure falls, and the RV pressure rises. Also, the ventricular pressure tracings show an early diastolic dip in pressure followed by a plateau phase due to rapid early diastolic filling and subsequent restriction in filling (square root sign or dip and plateau sign).

**Figure 4:**
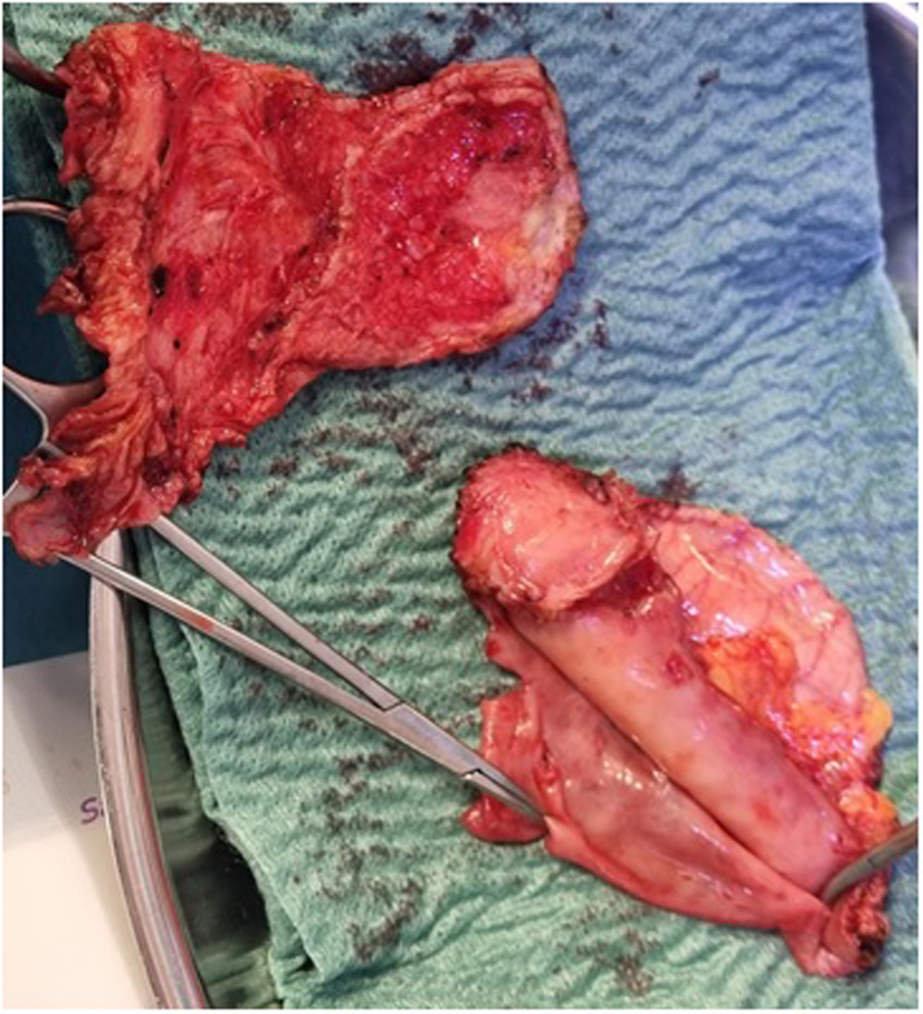
Specimen of anterior parietal layer pericardium post-pericardiectomy.

An 18-year-old male presented to our hospital with worsening fatigue. His past medical history was negative for any pathologies. He progressively developed pericardial and pleural effusions; subsequently, polyserositis was diagnosed. Therapy with ibuprofen, corticosteroids, and colchicine was started without any significant improvements; therefore, the patient was started on anakinra, which also did not contribute to any substantial improvement in the condition. Broad serological tests of infectious and collagen diseases were performed, including hepatitis viruses, toxoplasma, mycoplasma, venereal disease research laboratory (VDRL), cytomegalovirus, human herpes 6 (HH6), human herpes 8 (HH8), Epstein–Barr virus (EBV), human immunodeficiency virus, Coxsackie viruses, and antinuclear and rheumatic factors. All results were negative except for immunoglobulin M (IgM) antiviral capsid antigen (VCA) for EBV. Our patient was not related to a tuberculosis-endemic area; however, PCR for tuberculosis was performed in blood samples, sputum, and pericardial fluid, and the results were negative. Blood tests were found weakly positive for IgM VCA, and the extended autoantibody panel (ANA, ENA, ANCA, ASCA, and FR) was negative. Screening for celiac disease was also negative, and thyroid function tests were within normal limits. We also found tamponade pericardial effusion. It was, therefore, necessary to perform the first pericardiocentesis. Pericardial fluid was negative for malignant cells yet positive for inflammatory cells, and the polymerase chain reaction (PCR) test was positive only for EBV. Examinations for all other infectious agents (HH6, HH8, TB, CMV, *Enterovirus*) were negative. A chest computed tomography (CT) scan was performed, identifying lymph node conglobate in the right lung hilum, bilaterally in the supraclavicular and mesenteric areas. A bone marrow biopsy was also performed and was negative for lymphoproliferative disease. The transthoracic echocardiography showed moderate dysfunction of the left ventricle (LV) (with an ejection fraction of 46%), severe desynchrony of the ventricular septum, a global longitudinal strain (GLS) of −12.9%, the right ventricle (RV) with a normal function, and no significant regurgitation of the atrioventricular valve (Figure 1A). Cardiac magnetic resonance imaging (MRI) showed mild global biventricular systolic dysfunction [left ventricular ejection fraction (LVEF) 46%, right ventricular ejection fraction (RVEF) 47%] and circumferential pericardial effusion (maximum thickness 15–16 mm); right pleural effusion was identified (maximum thickness 3.5 cm), and there were no signs of pericardial constriction. Several days after pericardiocentesis with the extraction of about 400 ccs of blood serum liquid, a positron emission tomography (PET) CT scan and whole-body MRI scan were performed to exclude any underlying neoplastic processes. Once clinical and hemodynamic improvements were achieved, the patient was discharged with indications for close follow-up. One and two months after the patient was discharged, the echocardiography showed a stable condition with the persistence of a slight amount of pericardial effusion in the apical site and along the lateral wall of the left ventricle. After 3 months and mainly after 4 months, the echocardiogram showed worsening of the condition toward effusive-constrictive pericarditis, with enlargement of atrial sizes, alterations of diastolic function, dilation of the hepatic veins and inferior vena cava, and thickening and hyper-reflection of the pericardial leaflets. Abdominal ultrasound revealed effusion in all quadrants. A cardiac MRI was performed also 6 months after the first one and confirmed constriction with diffuse fibrotic thickening of the pericardial sac and signs of ventricular interdependence evident in the dynamic sequences (flattening of the interventricular septum in maximal inspiration), suggestive of constrictive physiology (Figure 2). The persistence of pericardial effusion along the mid-apical lateral wall of the LV was also identified (maximal thickness 10 mm). Finally, to confirm a restrictive picture, cardiac catheterization was performed, highlighting multiple elements suggestive of constrictive physiology with equalization of the diastolic filling pressure of the cardiac chambers and left and right ventricular diastolic “dip and plateau” appearance. Marked ventricular interdependence during the respiratory cycle and post-capillary pulmonary hypertension was noted. A diagnosis of constrictive effusive pericarditis was made (Figure 3, Table 1). The pre-operative cardiac catheterization showed pressure in the pulmonary artery of 35/21–25 mmHg and pulmonary wedge pressure of 22 mmHg (Table 1). At this point, the patient underwent anterior and posterior pericardiectomy surgery with preservation of the phrenic nerves, a procedure performed in normothermic cardiopulmonary bypass with a beating heart (Figure 4). Both intraoperative and postoperative periods were uncomplicated. The histological sample confirmed marked fibrous thickening with extreme rarefaction and fragmentation of the elastic fibers and mild lymphoplasmacytic inflammatory infiltrate (CD3+, CD20+, and CD38+), as well as neutrophilic granulocytes with numerous hemosiderin deposits and neovascularization. The *in situ* hybridization investigation with the Epstein–Barr-encoded small RNA (EBER) probe for the detection of EBV was positive, confirming the clinical suspicion of chronic pericarditis caused by EBV. Transesophageal echocardiography performed immediately after surgical excision of the fibrous pericardium showed complete recovery and normalization of ventricular septal motion. A few weeks after surgery, abdominal ultrasound showed a complete absence of peritoneal effusion. One week after surgery, transthoracic echocardiography was repeated and showed normalization of left ventricular function (3D ejection fraction 55%), and the GLS improved (−18.3%) (Figure 1B). The follow-up at 3, 6, and 12 months revealed no signs and symptoms related to diastolic heart failure, and echocardiogram findings were normal.

**Table 1:** Results of Cardiac catheterization

Pressures	Value
Left Ventricle	113/0-22 mmHg
Aorta	113/66-85 mmHg
Right Atrium	-/-/18 mmHg
Right Ventricle	45/12-22 mmHg
Pulmonary Artery	35/21-25 mmHg
Wedge Pressure	-/-/22 mmHg
**Saturations**	**Value**
Aorta	99%
Pulmonary Artery	71.3%
**Cardiac Index**	2.6 L/min/mq
**Transpulmonary pressure gradient **	3 mmHg
**Pulmonary vascular resistance index**	1,1 WU

## Discussion

As early as the 1950s, Miller et al. described the development of acute pericarditis during EBV infection (2). Pericardial involvement in infectious mononucleosis is variable and quite rare. Acute cases are described with positive responses to medical therapy without pericardiocentesis (3). However, cases of cardiac tamponade have been very rarely reported and were always limited to the acute phase without progression toward constrictive pericarditis (4). The probability that acute pericarditis may evolve into constrictive pericarditis is closely linked to etiology, being very low (<1%) in viral and idiopathic forms; intermediate (2%–5%) in immune-mediated conditions, as well as in neoplastic diseases of the pericardium; and high (20%–30%) in bacterial forms (5). In our case, the patient initially presented with symptoms of worsening fatigue and blood positivity for EBV VCA IgM antibodies. However, swollen lymph nodes were limited to the chest and were detected only after the chest CT scan. The progression to constrictive pericarditis was rapid. To our knowledge, this is the second reported case of constrictive effusive pericarditis due to EBV infection. In 1993, another case of a 42-year-old male with constrictive effusive pericarditis was described by Satoh et al. (6). In that case, liver damage secondary to the patient's alcohol abuse was hypothesized as a condition favoring the infection, which may have suppressed the patient's immune system, making him more prone to infection. However, this is not what was observed in our patient, who, on the other hand, was much younger, was involved in competitive sports, and was not using alcohol. Thus, we hypothesized that progression to the chronic form evolved from infectious mononucleosis. The presence of the viral genome demonstrated by PCR in the anatomical specimen unequivocally demonstrated the viral cause of constrictive pericarditis. The patient and family denied prior known EBV infections. For this reason, when the patient came to our observation for the first time, about 2 months after the onset of symptoms and 6 months before the definitive diagnosis of constrictive pericarditis, he had active mononucleosis which subsequently evolved into constrictive pericarditis. We think this is a good representation of this rare condition that should be considered as a differential diagnosis for constrictive pericarditis, especially when all the other more common causes of constrictive pericarditis are absent. Timely diagnosis and surgical treatment led to an immediate and marked improvement in the patient's clinical condition, allowing him to continue his sports at a competitive level.

## Data Availability

The raw data supporting the conclusions of this article will be made available by the authors, without undue reservation.
