# Corrigendum: Case Report: Epstein–Barr virus and constrictive pericarditis—an unusual combination

**DOI:** 10.3389/fped.2024.1390495

**Published:** 2024-03-07

**Authors:** Mario Panebianco, Eduard Limonjiani, Roberto Formigari, Veronica Bordonaro, Aurelio Secinaro, Enrico Cetrano, Adriano Carotti, Lorenzo Galletti, Sonia Albanese

**Affiliations:** ^1^ Clinic Area of Fetal Neonatal and Cardiologic Science, Bambino Gesù Children’s Hospital, IRCCS, Rome, Italy; ^2^ Advanced Cardiothoracic Imaging Unit, Bambino Gesù Children’s Hospital, IRCCS, Rome, Italy

**Keywords:** constrictive pericarditis, Epstein–Barr virus, ventricular mechanics, mononucleosis, effusive-constrictive pericarditis

A Corrigendum on Corrigendum: Case Report: Epstein–Barr virus and constrictive pericarditis—an unusual combination By Panebianco M, Limonjiani E, Formigari R, Bordonaro V, Secinaro A, Cetrano E, Carotti A, Galletti L and Albanese S. (2023) Front. Pediatr. 11:1215928. doi: 10.3389/fped.2023.1215928

In the published article, the authors’ names were incorrectly written as “M. Panebianco, E. Limonjani, R. Formigari, V. Bordonaro, A. Secinaro, E. Cetrano, A. Carotti, L. Galletti and S. Albanese”. The correct spelling is “Mario Panebianco, Eduard Limonjiani, Roberto Formigari, Veronica Bordonaro, Aurelio Secinaro, Enrico Cetrano, Adriano Carotti, Lorenzo Galletti, Sonia Albanese”.

The authors apologize for this error and state that this does not change the scientific conclusions of the article in any way. The original article has been updated.

